# Preterm Cord Blood Contains a Higher Proportion of Immature Hematopoietic Progenitors Compared to Term Samples

**DOI:** 10.1371/journal.pone.0138680

**Published:** 2015-09-29

**Authors:** Marina Podestà, Matteo Bruschettini, Claudia Cossu, Federica Sabatini, Monica Dagnino, Olga Romantsik, Grazia Maria Spaggiari, Luca Antonio Ramenghi, Francesco Frassoni

**Affiliations:** 1 Laboratorio Cellule Staminali Post-Natali e Terapie Cellulari, IRCCS Istituto Giannina Gaslini, Genoa, Italy; 2 Neonatal Intensive Care Unit, Department of Pediatrics, Institute for Clinical Sciences, Lund University, Lund, Sweden; 3 Dipartimento di Medicina Sperimentale, Università degli Studi di Genova, Genoa, Italy; 4 Neonatal Intensive Care Unit, IRCCS Istituto Giannina Gaslini, Genoa, Italy; French Blood Institute, FRANCE

## Abstract

**Background:**

Cord blood contains high number of hematopoietic cells that after birth disappear. In this paper we have studied the functional properties of the umbilical cord blood progenitor cells collected from term and preterm neonates to establish whether quantitative and/or qualitative differences exist between the two groups.

**Methods and Results:**

Our results indicate that the percentage of total CD34+ cells was significantly higher in preterm infants compared to full term: 0.61% (range 0.15–4.8) vs 0.3% (0.032–2.23) p = 0.0001 and in neonates <32 weeks of gestational age (GA) compared to those ≥32 wks GA: 0.95% (range 0.18–4.8) and 0.36% (0.15–3.2) respectively p = 0.0025. The majority of CD34+ cells co-expressed CD71 antigen (p<0.05 preterm vs term) and grew in vitro large BFU-E, mostly in the second generation. The subpopulations CD34+CD38- and CD34+CD45- resulted more represented in preterm samples compared to term, conversely, Side Population (SP) did not show any difference between the two group. The absolute number of preterm colonies (CFCs/10microL) resulted higher compared to term (p = 0.004) and these progenitors were able to grow until the third generation maintaining an higher proportion of CD34+ cells (p = 0.0017). The number of colony also inversely correlated with the gestational age (Pearson r = -0.3001 p<0.0168).

**Conclusions:**

We found no differences in the isolation and expansion capacity of Endothelial Colony Forming Cells (ECFCs) from cord blood of term and preterm neonates: both groups grew in vitro large number of endothelial cells until the third generation and showed a transitional phenotype between mesenchymal stem cells and endothelial progenitors (CD73, CD31, CD34 and CD144)The presence, in the cord blood of preterm babies, of high number of immature hematopoietic progenitors and endothelial/mesenchymal stem cells with high proliferative potential makes this tissue an important source of cells for developing new cells therapies.

## Introduction

In mammals, the initial phase of blood production occurs in yolk sac (YS) and is termed primitive haematopoiesis; during this phase, blood cells and large erythroid progenitors with megakaryocyte and macrophages potential can be detected, loosely associated with vascular endothelial cells (the so-called blood islands) [1]. These cells are rapidly replaced by embryonic definitive erythrocytes which are smaller than their primitive counterparts but larger than adult erythrocytes [2]. In humans, until 30 days gestational age (GA), the area surrounding the dorsal aorta, termed the Aorta-Gonad Mesonephros (AGM) region, consists in a matrix with CD146+ vascular endothelial cells capable to generate Haematopoietic Stem Cells (HSCs) with higher self-renewal capacity compared to UCB and adult BM HSCs[3,4,5]. The hematopoietic activity in the AGM region precedes that in the embryonic liver, which is colonized around week +7–8 of development and that in the placenta, which acquires HSC activity starting from week +9 of development, long term after the emergence of HSCs in the AGM region [6]. By week+11 well formed arterioles permit cell trafficking and migration and from week +16 hematopoiesis is organized within long bones with areas of fully calcified trabecular bones and areas of dense hematopoiesis [7].

The relative contribution of each of the above sites to the final pool of adult Hematopoietic Stem Cells (HSC) remains largely unknown and it is believed that none of these sites is accompanied by de novo HSC generation, rather, than these niches support the expansion of migrating HSCs capable of hematopoietic reconstitution, at least starting from AGM region [8].

HSCs may differ in their properties depending on their location (fetal liver, bone marrow, placenta) and on the age of the fetus; thus, it is important to identify mechanisms that regulate their cell function. The classical hierarchy diagram depicting progenitors arising from an “ideal” HSC provides a simplified view of the hematopoietic system. HSCs may be described more accurately as groups of cells with varying developmental potentials based on intrinsic properties due to their own genetic program and inputs from the cellular niches in which they reside [9].

Human cord blood (UCB) is a reservoir of a relatively high number of stem cells (HSC, EPC,HSC), with unique functional characteristics probably due to the fact that these cells are neither completely fetal nor adult cells.Its therapeutic use has been proved since 1988, when the first allogeneic HSC transplant was performed; currently, more than 160 public UCB banks have been established worldwide and there are ~730 000 UCB units available for public use, mainly for HSC transplant. In addition, an estimated 4.0 million UCB units have been stored for private or family use as guarantee of future application in regenerative medicine and cellular therapies [10].Despite the extensive characterization of UCB progenitors cells, few studies have examined the properties of preterm and early preterm cord blood cells. These studies have demonstrated that preterm UCB shows a significantly higher concentration of haematopoietic progenitors with increased clonogenic capacity compared to term and an early cellular differentiation, at least in vitro [11, 12, 13]. However, despite the higher proliferative potential, preterm human HSCs demonstrate an impaired homing ability into the NOD/SCID mice, probably due to a lower expression of adhesion molecules [14].

Preterm birth is associated with high mortality rate and high risk of developing disability. The number of long term complications like bronchopulmonary dysplasia (BPD), retinopathy of prematurity and intracranial hemorrhages, diseases associated to tissue damage, are inversely related to gestational age. Recent insights into pre-clinical studies have focused mainly on potential forms of treatment for BPD, the chronic lung disease of prematurity [15–17]. In fact, it has been demonstrated that the frequency of ECFCs is directly correlated with the gestational age and that extremely preterm infants, who display lower numbers at birth, have an increased risk of developing BPD due to lung vascular immaturity [18].In this contest, cord blood-derived stem cells may be a suitable adjuvant therapy for indications in which inflammation and tissue damage occurs.

In this paper, we have studied the functional properties of the umbilical cord blood cells collected from term and preterm neonates in order to establish whether quantitative and qualitative differences exist between the two groups. These cells may be a source of hematopoietic and non-hematopoietic progenitors for immature newborns, to be used for prevent complications related to inflammation, immune disregulation and organ fibrosis.

## Materials and Methods

### Samples

Cord blood was obtained from umbilical cord vessels of premature and full-term infants of pregnant women immediately after delivery. All participants provide their written informed consent to participate in this study.Exclusion criteria were positivity for HBV, HCV and HIV.

This study was approved by the Ethics Committee of the IRCCS Istituto G. Gaslini, Genoa (N° 164 22^th^ July 2013).The cord blood samples were transferred into the laboratory and analyzed within 12 hours from collection. The definitions of preterm and full term used herein are those adopted by the World Health Organization: <37 weeks gestational age (GA) and ≥37 weeks GA respectively; conversely, very preterm neonates are defined as having GA <32wks.

### Immunophenotypic analysis, side population assay, ALDH activity, and pluripotency gene expression

The enumeration of the hematopoietic progenitor cells (CD34+) was performed by FACS analysis as previously described [19]. After immunofluorescence staining, erythrocytes were lysed with ammonium chloride lysis solution, acquired within one hour by flow cytometry (FACSCANTO II, BD Biosciences) and analyzed using FlowJo software (TreeStar Inc., Ashland, OR, USA). CD34+ cells were gated according to the modified ISHAGE criteria [20].

Analysis of immature progenitors within CD34^+^ compartment was performed using antihuman antibodies: VioBlue-CD34 (MiltenyiBiotec), FITC- Lineage Cocktail 1 (lin 1; CD3, CD14, CD16, CD19, CD20, CD56), APC-CD38 (BD) and PE-CD90 (BD Biosciences)[12]

Side population (SP) cells were identified in both preterm and term CB as previously described [21]. Mononuclear cells were resuspended at 10^6^ cells/mL in warmed staining buffer [DMEM supplemented with 2% fetal calf serum (FCS)] and incubated with 5 μM Dye Cycle Violet (DCV; Molecular Probes Invitrogen Inc.,) for 30 min at 37°C. To set the gates for SP cells, an aliquot of cells was pre-incubated with 50 μM Verapamil (Sigma-Aldrich) for 30 min at 37°C before staining with DCV. At the end of the incubation, cells were labeled with anti-CD34 (BD Biosciences), CD338 (ABCG-2; Miltenyi Biotechnology) and 7-AAD (EBioScience) and subsequently detected on an LSRFortessa flow cytometer (BD Biosciences). Results wereanalyzed using FlowJo software according to the gating strategy previously described [22].

Aldehyde dehydrogenase (ALDH) activity in HSC was evaluated by using Aldefluor reagent (StemCell Technologies) according to the manufacturer’s instructions. Cells were resuspended in aldefluor assay buffer and an appropriate amount of aldefluor substrate was added to 1x10^6^ MNCs. Cells were incubated for 30 minutes at 37°C for conversion of substrate to a fluorescent product. An aliquot of Aldefluor- stained cells were immediately treated with diethylaminobenzaldehyde (DEAB), a specific ALDH inhibitor, to serve as negative control. Aldefluor- substrate labeled cells were then costained with anti- CD34 VioBlue (Miltenyi Biotechnology) and CD38 APC (Bd Biosciences) and subsequently analyzed by FACS.

To evaluate pluripotency gene expression, CD34- stained MNCs were fixed and permeabilized with BD Cytofix/Cytoperm kit (BD Biosciences) according to the manufacturer’s instructions. Cells were stained for Nestin, OCT3/4 and Nanog with an PE-conjugated mAb(BD Pharmigen, EBioScience, BD Pharmigen) or isotype controls for 20 minutes at 4°C and then analyzed by flow cytometry.

### Clonogenic assays

Methylcellulose-based semisolid assay was used to evaluate Colony Forming Cells (CFCs) [23]. All assays (first and secondary generation colonies) were performed using Methocult H4434 Classic (StemCell^TM^ Technologies, Vancouver BC) which is a methyl-cellulose based medium added with rhSCF, rhGM-CSF,rhIL-3 and rhEPO for the culture of human cells.

*First generation*: 5μl of whole Cord Blood (WCB) were added to 1 mL semisolid culture medium and plated in duplicate on 24-wells plates. After 14 days of incubation in a humidified atmosphere at 37°C and 5% CO_2_, colonies were evaluated for number and morphology in the same dish using an inverted microscope.*Second generation*: colonies grown in first generation were collected, pooled and dissociated using Dulbecco’s modified Eagle’s medium(DMEM,Life Technologies). Nucleated cells (NC) were counted manually and 5x10^4^NC were plated using the same culture conditions of the first generation. The same procedure was performed for the third generation culturing 10^5^ NC.

The enumeration of CD 34+ cells was performed by FACS analysis on cells obtained from colony pools(first and second generation).

### CD34 positive cell enrichment and RNA isolation

UCB mononuclear cells (MNC) were isolated from fresh heparinized cord blood by density gradient separation and enriched for CD34^+^ antigen by immune-selection method (Miltenyi Biotech, Germany). Following two cycles on separate columns, CD34^+^cell purity was assessed by flow cytometry: only samples ranging between 85% to 95% were selected for RNA extraction.

Isolation of total RNA from CD34^+^ cells was performed with the RNeasyMicroKit (Qiagen AG, Hilden, Germany). RNA obtained was reverse transcribed using the High Capacity RNA-to-cDNA Kit according to the manufacturer’s instructions and performed on 2720 thermal cycler (Applied Biosystems). RNA isolated from different samples (2–4) was pooled to obtain the amount requested for gene profiling.

### TaqMan Array Gene Profiling

TaqMan® Gene Expression Assays have been designed using manufacturer’s validated bioinformatics pipeline (Applied Biosystems, CA, USA) and consisted in a selected list of target genes (S1 Table) which were processed as described by the instructions. Quantitative real-time PCR (qPCR) was performed with the ViiA™ 7 Real-Time PCR System (AppliedBiosystems). Relative quantification was established by Comparative Ct method (2^−ΔΔCt^) with 18S ribosomal RNA as internal control and normalized to the expression of Human Reference Total RNA (Stratagene). Analysis of the results was done using the Expression Suite Software V1.0.3 (Life Technologist).

### Endothelial Colony Forming Cell (ECFC)

ECFC cultures were performed by plating UCB-MNCs (5x10^6^/well) on 6-wells culture dishes coated with collagen (Sigma-Aldrich, Milan Italy) in endothelial cell growth medium EGM-2 Bullet Kit (Lonza-Euroclone) [24]. ECFCs are expressed as ECFCs frequency/10^7^MNC. Endothelial colonies were identify using a) morphological criteria; b) immunophenotypic analysis (fluorescence microscopy and flow cytometry).

For immunofluorescence analysis, endothelial colonies were fixed with 4% paraformaldehyde, washed with phosphate-buffered saline (PBS) and then incubated with mouse anti-human CD31 (DakoCytomation, Milan, Italy).Goat anti mouse-Alexa Fluor 488 (Molecular Probes, Invitrogen S.R.L., Milan, Italy) conjugated antibodies was used as secondary antibodies and4’,6’-diamidino-2-phenylindole (DAPI; Vector Laboratories, Burlingame,CA, USA) was used for nuclear counterstaining. Both cell morphology and green fluorescence were then analyzed by inverted fluorescent microscope (Axio Vert. A1, Carl Zeiss S.p.A., Milan Italy)

Immunophenotypic characterization was performed on expanded ECFC at P2–P3. The following monoclonal antibodies were used: CD14 and anti-CD45 (BD Biosciences) anti CD-31, anti-CD90 and anti-CD144 (BD Pharmigen), anti-CD34, anti CD73, anti-CD105, anti- CD133, anti-CD146 and anti-CD309 (KDR;MiltenyiBiotec). Stained cells were acquired by a MACSquant^®^ Analyzer 10 (MiltenyiBiotec) and the multicolor analysis was performed using FlowJo software.

### Statistical Analyses

Statistical analyses were performed using the GraphPad Prism 3.0 statistical platform.

The Mann-Whitney test was used to compare the differences between preterm and term biological samples. All differences with p<0.05 were considered statistically significant. Data were expressed as median (range) for all the statistical analysis. Correlation analysis was performed using Pearson’s Correlation r.

## Results

### Samples

Our study included 58 term (1 twin) and 50 preterm (11 twins) UCB samples; in the preterm group, 21/50 neonates had a gestational age of <32 wks.

Clinical data of pre-term infants, including birth weight, gestational age, gender, clinical problems after delivery and survival outcome are presented in Table 1. Preterm neonates had a lower number of white blood cells (WBCx10^3^/μL): median 4.7 (2.2–7.5) vs 8.3 (5.0–15.5) (p = 0.0005) in comparison to full term.

**Table 1:** Clinical data of preterm neonates.

Group (GA wks)	BW (gr)	GA (wks)	Gender (M/F)	Twins (N)	Delivery Caesarean /Vaginal	RDS (%)	Survival (%)	Cause of death (N)	EOS (%)
< 32 (n = 21)	1120 (600–1915)	28 (22–31)	15/6	3	19/2	95.2	90.5	Thyroid tumor (1) Oligohydramnios (1)	14.3
>32(n = 29)	2085 (1370–3180)	34(32–36)	17/12	8 (1 triplet)	22/7	58.6	100		0

n = number of cord blood samples; GA,gestational age; BW, body weight; EOS,early onset sepsis; RDS, neonatal respiratory distress Syndrome; wks, weeks. Values are represented as median (range).

### CD34

**Fig 1:**
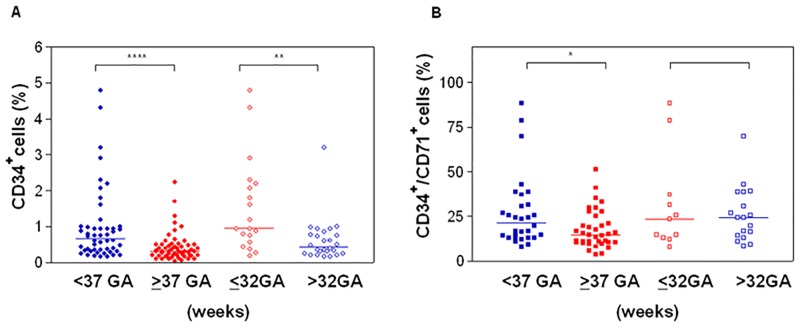
Frequency of circulating CD34+ cells in UCB. Four groups are considered based on gestational age (GA) of the neonates. (A) Percentage of total CD34+ cells. (B) Percentage of CD34+CD71+. *P<0.05; **P<0.01; ****P<0.0001

We assessed whether preterm UCB leads to changes in the proportion of circulating total CD34^+^ cells. The percentage of total CD34^+^ cells inversely correlated with the gestational age: 0.61% (range 0.15–4.8) in preterm infants (n = 50) and 0.3% (0.032–2.23) in full term (n = 57) p = 0.0001as well as in neonates <32 weeks of gestational age compared to those having 32–36 wks GA: 0.95% (range 0.18–4.8) and 0.36% (0.15–3.2) respectively p = 0.0015. Similarly, the number of CD34^+^ CD71^+^ cells resulted higher in preterm UCB (n = 30) with respect to term ones (n = 37): 24.35% (7.8–42.9) vs 14.5% (1.5–51.2) p = 0.021 but no differences were detectable in very preterm compared with samples from neonates between 32 and 36 wks of gestational age. (Fig 1) Different subpopulations were studied to establish the proportion of more immature progenitors within CD34^+^ compartment: no difference was found in the percentage of CD34^+^ CD90^+^ CD45RA^-^, but CD34^+^ CD38^-^ and CD34^+^CD 45^-^cells resulted more represented in samples from pre-term cord blood than in full term (Table 2). Finally, the ALDH activity and the expression of stemness genes like Nestin, Nanog and Oct3/4 did not differed in CD34+ cells from term and preterm samples.(S1 Fig).

**Table 2:** Different CD34+ subpopulation in preterm and term cord blood.

CD34 Subpopulation (%)	Preterm (n = 6)	Term (n = 6)	^a^P-value
CD34+CD38^-^	65.1 (37.1–84.3)	31.1 (8.9–35.3)	<0.001
CD34+CD45^-^	0.12 (0.09–0.14)	0.02 (0.00–0.04)	0.041
CD34+CD90^+^/CD45RA^-^	1.54 (1.05–3.21)	2.19 (0.52–14.50)	0.309

n. = number of cord blood samples Values are represented as median (range)

^a^ *p* values were calculated using Mann-Whitney t test.

### Side population

**Fig 2:**
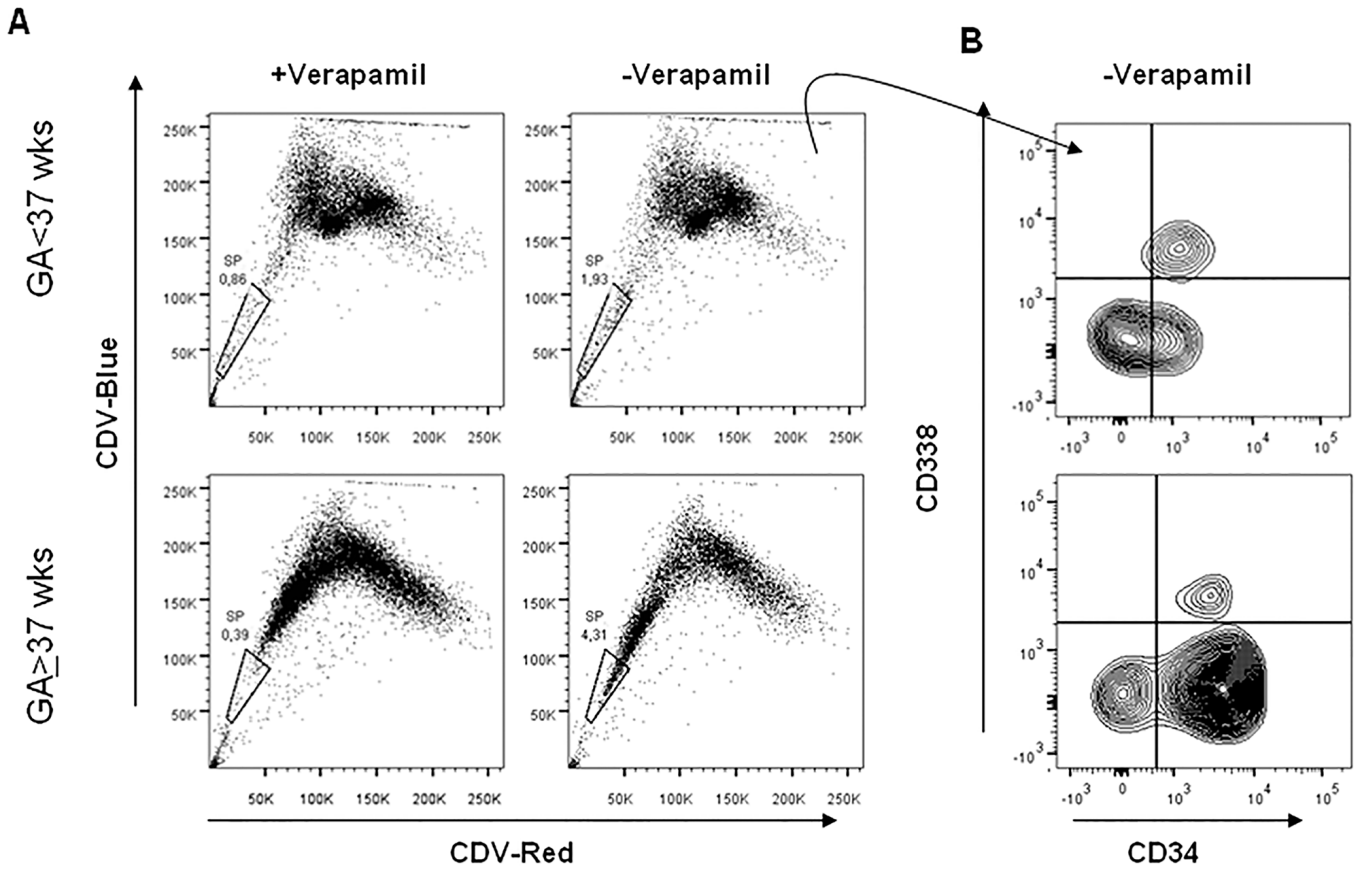
Side population (SP) analysis of human mononuclear cells obtained from pre-term and term cord blood. (A) DyeCycle Violet (DCV) blue *vs* red fluorescence is shown for all cells within the viable (7AAD-) CD45+dim/SCC low gate of the scatter plot. In these representative samples, 1.93% (for pre-term: GA<37 wks) and 4.31% (for term: GA≥37 wks) of the cells fall within the gated SP (-Verapamil) and are sensitive to Verapamil (+ Verapamil). (B) Expression of CD34 and CD338 (ABCG2 transporter) within SP population of preterm (n = 5) and term (n = 5) cord blood.

Preterm (n = 5 samples) and term (n = 5 samples) cord blood were tested for SP cells. No differences were found between the two groups: median 1.9 (range 1.15–9.6) and median 4.3 (range 0.52–16.9)(p = 0.78). Conversely a significant difference was observed in the distribution of CD34-/CD34+ cellswithin SP: in fact preterm SP showed higher percentage of CD34- cells compared to term SP: median 45.9 range28.8–59.0 vs median 18.8 range 10.2–24.6 (Mann-Whitney test p = 0.03). (Fig 2)

### Colony number

The absolute number of total CFCs /10μL WCB as well asthe frequency of CFC/5x10^4^NC observedin the second generationresulted higher in preterm samples compared to term ones (p = 0.004 and p = 0.05 respectively; Table 3). In addition,the CFCs number /10μL WCBresulted inversely correlated with the gestational age of the neonates (Pearson r = -0.3001 p = 0.0168), although the number of cells for each colony was not affected by gestational age (p = 0.9).

**Table 3:** Clonogenic growth efficiency in preterm and term infants.

GENERATION									
FIRST					SECOND				THIRD
Group	Cell number per colony/x10^5^NC	CFC/ 10microLWCB	BFU-E/ 10microL WCB	CD34+ (%)	Group	CFC/5x10^4^ NC	BFU-E/ 5x10^4^ NC	CD34+ (%)	CFC/ 10^5^NC
Preterm (n = 33)	1.1 (0.39–1.92)	68 (36–138)	28 (10–60)	3.1 (1.2–5.2)	Preterm (n = 14)	21 (0–88)	18 (0–86)	8.4 (5.0–10.5)	2 (0–3)
Term (n = 35)	1.0 (0.4–2.1)	60 (8–84)	30 (2–62)	5.3 (3.6–5.3)	Term (n = 16)	5 (0–75)	3 (0–32)	0.8 (0.3–3.0)	0
^a^P-value	0.9	0.004	0.9	0.17	^a^P-value	0.05	0.002	0.0017	

n = number of samples; CFC,Colony forming cells; BFU-E, burst forming units of the erythroid lineage; NC, nucleated cells. Values are represented as median (range). first generation = colonies grown from whole cord blood (1); second generation = colonies grown from cells harvested pooling first generation colonies(2); third generation = colonies grown from (2)

^a^ *p* values were calculated using Mann-Whitney t test;

All lineages were represented in culture with a prevalence of erythroid colonies (BFU-E) in the first and in the second generation (Table 3). The re-plating efficiency of term CB resulted lower compared to preterm (68% vs 82% in secondary generation) and only pre-term samples (17%) could grow until the third generation.

In this contest, we have analyzed the percentage of CD34^+^ cells in colonies grown in semi-solid medium. No difference was observed between term and preterm UCB in the percentage of CD34^+^ cells in the first generation: median 5.28% (3.58–5.29) vs 3.05% (1.15–5.2) p = 0.17; conversely in the second generation, the median percentage of CD34^+^ resulted significantly higher in preterm colonies vs term: 8.39 (5.0–10.5) and 0.82 (0.32–3.0) respectively (p = 0.0017).

### Endothelial cells

**Fig 3:**
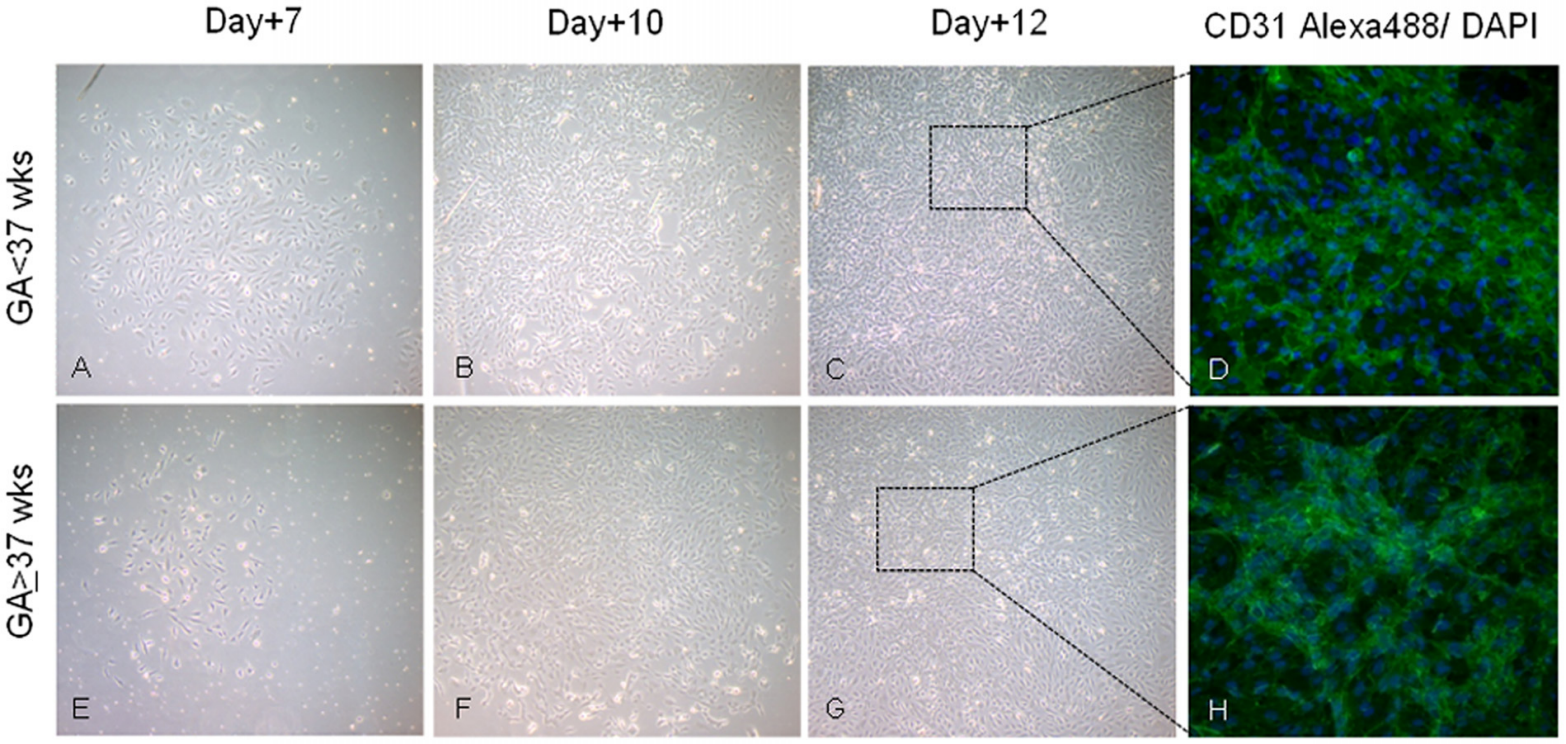
Endothelial Colony Forming Cell (ECFC) in pre-term and term- umbilical cord blood. Representative photomicrographs of ECFCs from mononuclear cells isolated from pre-term (GA<37 wks, A-C) and term (GA≥37 wks, E-G) cord blood at different time of culture (Original magnification 50X). (D,H) Immunofluorescence staining of CD31 (green) and DAPI (blue) (Original magnification 200X).

Colonies of endothelial cells (ECFCs) typically appeared between day +5 and day +14 and were identified as well-circumscribed monolayers of cobblestone-appearing cells which enlarged rapidly. These cells omogenously expressed CD31 antigen. A time-course picture of living ECFCs are show in Fig 3.

ECFCs showed in vitro a variable capacity to grow (11% UCB samples of both groups did not grow endothelial cells). The median frequency of ECFCs in cord blood samples of term and preterm neonates was7.8 /10^7^ MNC (range = 0–21.4) and 5.4 /10^7^(range 0–55.0), respectively (p = 0.5). This frequency does not correlate with the gestational age nor with other maternal (hypertension, antenatal steroid exposure, multiple pregnancy) or neonatal characteristics (sex, RDS, intubation, early-onset sepsis).

Endothelial progenitors gave rise to large colonies (median 2.0 x10^4^ nucleated cells/colony; range 0.3–6.1x10^4^) and expanded rapidly in culture: a median of 3.0 fold expansion in the number of endothelial cells from the first to the second generation (range 1.3–10.4) and a median of 2.6 fold expansion from the second to the third (range 0.9–8.4). No differences were observed between preterm and full-term CB (data not shown). ECFCs expressed typical endothelial markers (CD31, CD144, CD146) and CD73 (Table 4).

**Table 4:** Analysis of the cell surface antigens of cord blood ECFCs from preterm and term neonates.

Cell surface antigen	Preterm (n = 12)	Term (n = 12)
CD45	<10.0	<0.5
CD14	<1.0	<0.5
CD31	>95.0	>95.0
CD144	>95.0	>96.2
CD309	>29.4	>25.9
CD146	>94.9	>99.0
CD73	>98.7	>89.62
CD105	>99.0	>99.67
CD117	>50.0	>49.5
CD133	>32.3	>19.5

n. = number of samples evaluated.

Values are represented as percentage (%) of positive cells.

CD34 antigen was present in the majority of the endothelial cells (> 75%) expanded in vitro and showed a range of expression from bright to dim both in preterm and term samples. A small percentage of CD34^+^KDR^+^ cells were also represented in ECFCs from both preterm and full term CB (Table 5).

**Table 5:** Different CD34^+^ subpopulation in endothelial progenitors.

CD34 Subpopulation	Preterm (n = 5)	Term (n = 5)	^a^P-value
CD34^bright^	31 (24–60)	12.7 (2.1–58.2)	NS
CD34^dim^	65 (45–71)	60.7 (46.5–81.2)	NS
34+ tot.	73.4 (36–89.9)	85.2 (52–97)	NS
34+KDR+	8.1 (6.0–18.0)	6.74 (1.2–26.3)	NS

n. = number of samples evaluated; NS = not statistically significant. Values are represented as median (range).

^a^ *p* values were calculated usingMann-Whitney t test

### TaqMan® Gene Expression Assays

We have analyzed the expression of several genes, mainly related to stemness maintenance and cell differentiation in selected CD34+ cells (S1 Table). Overall, the pattern of expression did not significantly differed between the two groups. Only a few genes were not expressed in full-term CD34+ cells respect to preterm: NFIL3, ETS1, SET, PRDM1, and ID2. Several genes resulted differently over-expressed (as genes involved in the maintenance of self-renewal, stem cell reprogramming and pluripotency as well as genes essential in regulating the development and maintenance of hematopoiesis) in full term and in preterm progenitors but comparison between the two groups failed to identify statistically significant differences (S2 Fig).

## Discussion

In this study, we show that the proportion of total CD34^+^ cells in the cord blood of preterm neonates is higher compared to full term neonates and this difference is inversely correlated with gestational age: in fact, the highest proportion of CD34^+^ cells is found in samples from very immature babies (<32GA).There are additional evidences for the presence of a more immature progenitor in the preterm UCB compared to term: a) higher percentage of cells with the phenotype of immature HP (CD34^+^/CD38^-^);b) higher absolute number of CFCs; c) capacity to grow colony in vitro until the third generation; d) the significant inverse correlation between the absolute number of colony and the gestational age; e) higher proportion of CD34^+^ cells harvested from the second generation of colonies. All these features are paralleled by over-expression of genes involved in the maintenance of self-renewal (ID2, SET, TBPL1)[25–27] and genes related to the expansion of the hematopoietic stem cell pool (HOXB3, DPPA4) [28, 29].

We found that the majority of CD34+ cells co-express CD71 antigen (p<0.05 term vs preterm); this translate in a prevalent growth in vitro of large bursts [30]. Interestingly, in the second generation the vast majority of the colonies are erythroid. The prevalence of the erythroid lineage commitment within the CD34^+^ cell population and the capacity of these progenitors to growin secondary generation, is suggestive for the presence in cord blood of an immature progenitor devoid to transport oxygen, carbon dioxide and to facilitate vascular remodeling. These progenitor cells disappear very soon after birth[31, 8].

UCB from full term newborns exhibit a higher WBC count than UCB from preterm, but a lower HP concentration in keeping with a previous study [11].This difference is inversely correlated with the gestational age, thus suggesting that circulating CD34^+^ cells do not substantially expand from the second to the third trimester of the fetal life or that this population undergoes expansion but leaves the circulation and progressively reaches the hematopoietic niches in the bone marrow. In fact, it has been demonstrated in the humans, that the vascular microenvironment reaches relative maturity by 20^th^ gestational week and that osteoblastic/endosteal niche is completed later, when mesenchymal (endochondral) tissue became ossified[8]. Thus, it is possible that, a population of immature HP can persist into the blood stream of the preterm neonates before homing into a transient endothelial niche and finally into the trabecular bone, as demonstrated in the zebrafish model [32].

In our analysis, endothelial progenitor cells frequency (measured as ECFC) present in the cord blood did not show to be correlated with gestational age nor with maternal or neonatal characteristics. These data differ from other studies [18] which suggest that circulating endothelial progenitors are correlated with Bronchopulmonary Dysplasia (BPD) occurrence in preterm infants. These differences may be explained by the assay used: our data are based on functional studies and not on phenotypic analyses of whole cord blood.

ECFC grew in vitro large number of endothelial cells until the third generation that strongly expressed CD73, an ecto-5’-nucleotidase that convert adenosine triphosphate to adenosine, as well as CD31, CD34 and CD146, thus resembling a transitional phenotype between mesenchymal stem cells and endothelial progenitors. Usually, Endothelial-Mesenchymal Transition (EndMT) is considered a source for fibroblasts and myofibroblasts for cardiac development as well as, a pathologic condition occurring during tumor development and organ fibrosis. However, recent studies have found that EndMT cells also had ‘stemness’, which means they could differentiate into cells of multiple lineages [33]. In this contest, endothelial progenitors isolated and expanded in vitro from UCB, probably identify a populations of progenitors with high proliferative potential, immature phenotype (CD34^+^ KDR^+^CD45^-^) and capable to differentiate toward endothelial cells, smooth muscle cells and fibroblasts [34].

Cell therapy is a promising opportunity of regenerative medicine that can be used to treat some complications related to prematurity as respiratory distress, neurological, gastrointestinal and ophthalmological diseases [17]. However, the therapeutic use of preterm cord blood has some limits: the first one is that it is considered “not elegible for donation” due to the scarce number of cells except in the autologous setting [35]. The second is that preterm CD34+ cells show impaired homing ability, at least in the NOD/SCID mouse model [14]; these data do not still exclude that the re-infusion of hemopoietic progenitors with high proliferative potential, although not supporting a stable engraftment, may reduce the number of red cell transfusions in preterm babies [36].In addition, as in the paper [14] mice were transplanted using enriched CD34+ cells, it may be possible the fraction CD34- of the SP can have a role to ameliorate the hematological parameters.

Finally, the presence, in the cord blood of preterm babies, of endothelial/mesenchymal stem cells with high proliferative potential makes this tissue an important autologous source of cells capable to migrate to the site of inflammation for healing damage tissues [37] or to produce microvesicles which can protect organs from oxidative stress [38].

However, pre-clinical studies should clarify which therapeutic strategy may be more beneficial to prevent complications related to preterm birth: whether to infuse autologous fresh cord blood cells soon after delivery or to isolate and expand in vitro specific progenitor cells (HSC and/or endothelial) and re-infuse them at later time.

## Supporting Information

S1 Fig Expression of pluripotency genes and ALDH functional activity in cord blood CD34^+^ cells from preterm and term neonates. (A) A representative flow cytometric analysis of Nestin, OCT3/4, and NANOG expression by CD34^+^ cells from preterm (GA<37 wks) and term neonates (GA≥37 wks). The gated area represents the CD34^+^ cells among CB MNC. The black peak denotes isotype control. (B) ALDH expression in CB MNC. Gated ALDH+ cells (left panel) were evaluated for coexpression of CD34 and CD38 (right panel). One of 4 representative sample is shown.
(TIF)

S2 Fig Gene expression in cord blood CD34+ cells from preterm and term infants. CD34^+^ cells were purified from preterm (red bars) and term (blu bars) cord blood (CB). cDNA was prepared from total RNA and analysed as described in Materials and Methods. (A) Expression of gene found to be down-regulated in both preterm and term CB. (B) Quantitation of gene involved in self-renewal, stem cell reprogramming and pluripotency. (C) Quantitative analysis of genes related to maturation and commitment of T and B lineages (D) Quantitation of gene essential in regulating the development and maintenance of hematopoiesis and endothelial/hemopoietic transition. Fold change of mRNA expression by CD34^+^ cells purified from preterm was calculated by normalizing to the level of calibrator gene. The evaluation included n = 3 samples from both preterm and term CD34+ cells. sample was analysed in duplicate and contained a pool of CD34isolated from 2 to 4 CB from each group.
(TIF)

S1 Table List of target genes evaluated in CD34^+^ cells isolated from preterm and term cord blood. (DOCX)
