# Supplementary figures and images for: Preterm Cord Blood Contains a Higher Proportion of Immature Hematopoietic Progenitors Compared to Term Samples
Source: PLoS One. 2015 Sep 29;10(9):e0138680. doi: 10.1371/journal.pone.0138680 (PMC4587939; doi:10.1371/journal.pone.0138680)

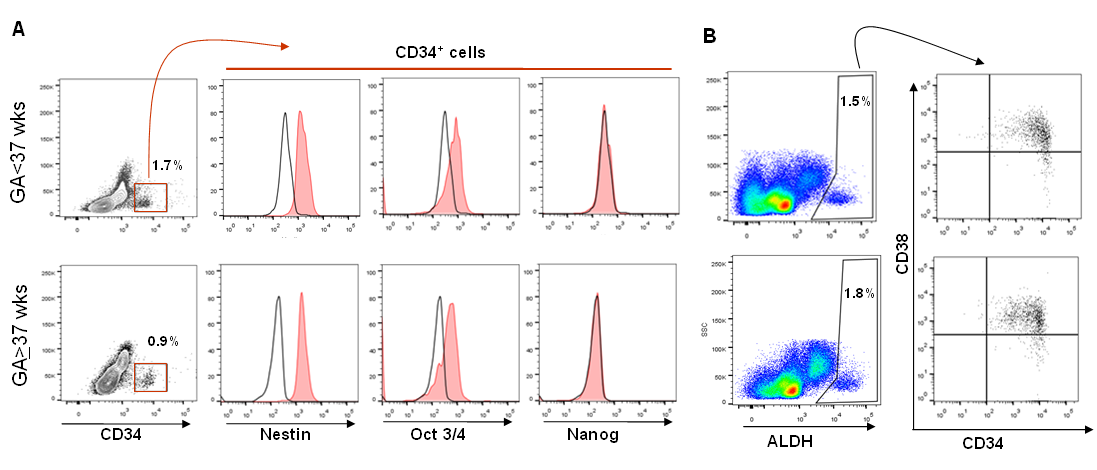

Supplement: S1 Fig — (A) A representative flow cytometric analysis of Nestin, OCT3/4, and NANOG expression by CD34+ cells from preterm (GA<37 wks) and term neonates (GA≥37 wks). The gated area represents the CD34+ cells among CB MNC. The black peak denotes isotype control. (B) ALDH expression in CB MNC. Gated ALDH+ cells (left panel) were evaluated for coexpression of CD34 and CD38 (right panel). One of 4 representative sample is shown. (TIF) [file pone.0138680.s001.tif]

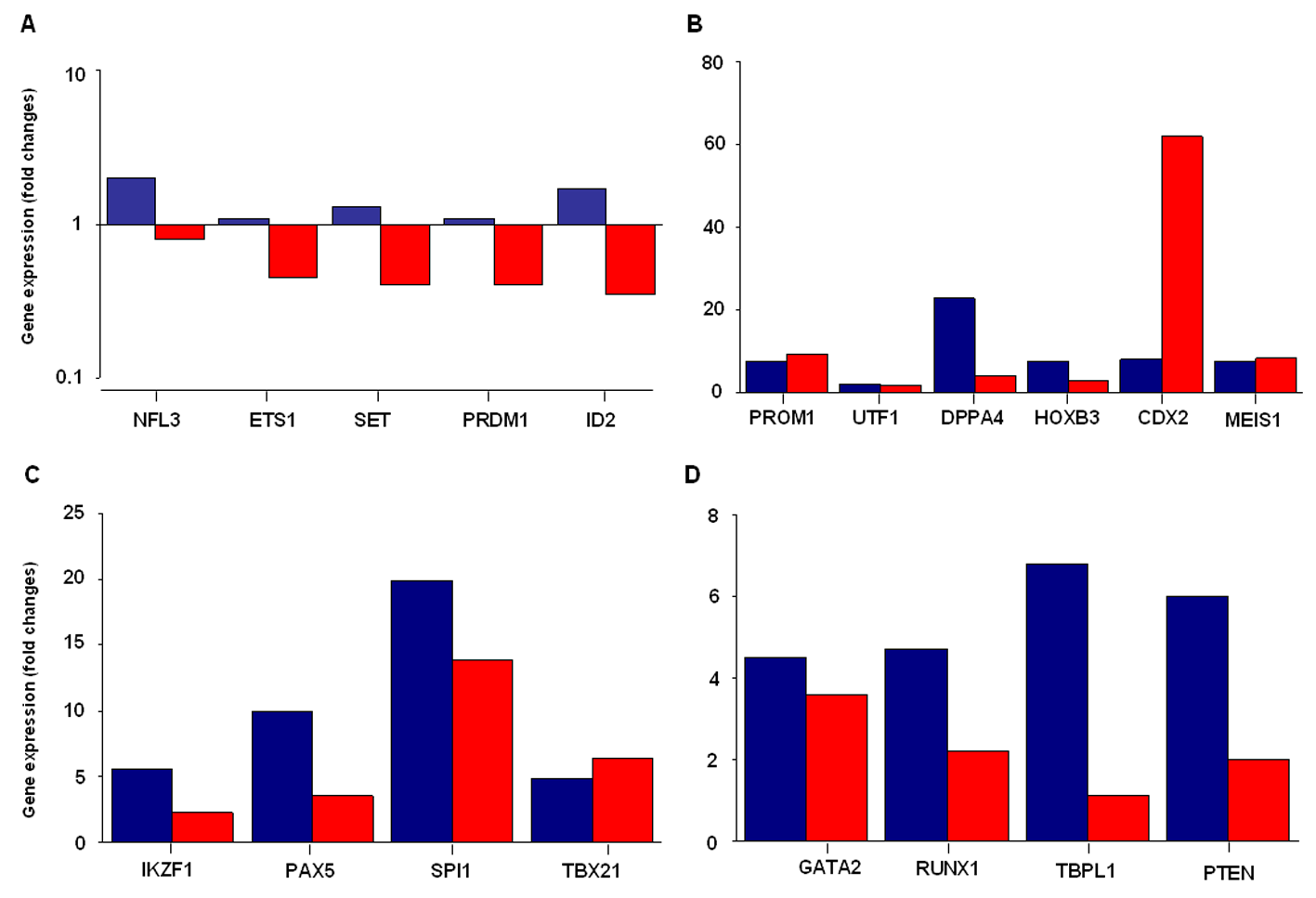

Supplement: S2 Fig — CD34+ cells were purified from preterm (red bars) and term (blu bars) cord blood (CB). cDNA was prepared from total RNA and analysed as described in Materials and Methods. (A) Expression of gene found to be down-regulated in both preterm and term CB. (B) Quantitation of gene involved in self-renewal, stem cell reprogramming and pluripotency. (C) Quantitative analysis of genes related to maturation and commitment of T and B lineages (D) Quantitation of gene essential in regulating the development and maintenance of hematopoiesis and endothelial/hemopoietic transition. Fold change of mRNA expression by CD34+ cells purified from preterm was calculated by normalizing to the level of calibrator gene. The evaluation included n = 3 samples from both preterm and term CD34+ cells. sample was analysed in duplicate and contained a pool of CD34isolated from 2 to 4 CB from each group. (TIF) [file pone.0138680.s002.tif]
