# Supplementary material for: Preterm Cord Blood Contains a Higher Proportion of Immature Hematopoietic Progenitors Compared to Term Samples
Source: PLoS One. 2015 Sep 29;10(9):e0138680. doi: 10.1371/journal.pone.0138680 (PMC4587939; doi:10.1371/journal.pone.0138680)
Supplement: S1 Table — (DOCX) [file pone.0138680.s003.docx]

**S1 Table. List of target genes evaluated in CD34^+^ cells isolated from preterm and term cord blood.**

| **Gene** | **Description** |
| --- | --- |
| TAL1-Hs01097987_m1 | T-cell acute lymphocytic leukemia 1 |
| GATA2-Hs00231119_m1 | GATA binding protein 2 (erythroid development) |
| EZH1-Hs00940463_m1 | enhancer of zeste homolog 1 (Drosophila) (in Human safeguards ES identity) |
| EZH2-Hs00544833_m1 | enhancer of zeste homolog 2 (Drosophila) |
| LIN28A-Hs00702808_s1 | lin-28 homolog A (C. elegans) |
| LIN28B-Hs01013729_m1 | lin-28 homolog B (C. elegans) (HP reprogramming to fetal hematopoiesis) |
| NANOG-Hs04260366_g1 | Nanog homeobox |
| NES-Hs04187831_g1 | nestin |
| VIM-Hs00185584_m1 | vimentin |
| POU5F1B;POU5F1;POU5F1P3-Hs00999634_gH | POU class 5 homeobox 1B,POU class 5 homeobox 1,POU class 5 homeobox 1 pseudogene 3 |
| PTEN-Hs02621230_s1 | phosphatase and tensin homolog (tumor-suppressor gene). |
| SOX1-Hs01057642_s1 | SRY (sex determining region Y)-box 1 |
| SOX2-Hs01053049_s1 | SRY (sex determining region Y)-box 2 |
| DPPA2-Hs00414521_g1 | developmental pluripotency associated 2 |
| HOXB3-Hs01587922_m1 | homeobox B3 (maintenance of self-renewal, proliferation) |
| FUT4-Hs01106466 | fucosyltransferase 4 (alpha (1,3) fucosyltransferase, myeloid-specific |
| SOX17-Hs00751752_s1 | SRY (sex determining region Y)-box 17 |
| JUNB-Hs00357891_s1 | jun B proto-oncogene |
| UTF1-Hs00864535_s1 | undifferentiated embryonic cell transcription factor 1 (maintenance of self-renewal) |
| ZFP42-Hs01938187_s1 | zinc finger protein 42 homolog (mouse) |
| PROM1-Hs01009250_m1 | prominin 1 (stemness maintenance) |
| FGF4-Hs00999691_m1 | fibroblast growth factor 4 |
| RUNX1-Hs00231079_m1 | runt-related transcription factor 1 (neoangiogenesis, endot./mes. Transition) |
| CDX4-Hs00193194_m1 | caudal type homeobox 4 |
| ITGB1-Hs00559595_m1 | integrin, beta 1 (fibronectin receptor, beta polypeptide, antigen CD29 includes MDF2, MSK12) |
| DPPA4-Hs00216968_m1 | developmental pluripotency associated 4 (maintenance of self-renewal) |
| DPPA3-Hs01931905_g1 | developmental pluripotency associated 3 |
| DPPA5-Hs00988349_g1 | developmental pluripotency associated 5 |
| DNMT1-Hs00945875_m1 | DNA (cytosine-5-)-methyltransferase 1 |
| DNMT3B-Hs00171876_m1 | DNA (cytosine-5-)-methyltransferase 3 beta |
| EHMT2-Hs00198710_m1 | euchromatic histone-lysine N-methyltransferase 2 |
| MLL-Hs00610538_m1 | myeloid/lymphoid or mixed-lineage leukemia (trithorax homolog, Drosophila) |
| MLL4-Hs00207065_m1 | myeloid/lymphoid or mixed-lineage leukemia 4 |
| SET-Hs00853870_g1 | SET nuclear oncogene (ES development, control of transcription) |
| SETD1A-Hs00322315_m1 | SET domain containing 1A |
| SETBP1-Hs00210209_m1 | SET binding protein 1 |
| SETD2-Hs01014784_m1 | SET domain containing 2 |
| SETD1B-Hs00902716_m1 | SET domain containing 1B |
| SETD5-Hs00216962_m1 | SET domain containing 5 |
| SETD6-Hs00227507_m1 | SET domain containing 6 |
| SETD7-Hs00363902_m1 | SET domain containing (lysine methyltransferase) 7 |
| SETD8-Hs01029949_m1 | SET domain containing (lysine methyltransferase) 8 |
| SETMAR-Hs00740846_m1 | SET domain and mariner transposase fusion gene |
| SMYD3-Hs00224208_m1 | SET and MYND domain containing 3 |
| SMYD4-Hs00736236_m1 | SET and MYND domain containing 4 |
| CTNNB1-Hs00355049_m1 | catenin (cadherin-associated protein), beta 1, 88kDa |
| FRZB-Hs00173503_m1 | frizzled-related protein |
| DVL1-Hs00182896_m1 | dishevelled, dsh homolog 1 (Drosophila) |
| MPL-Hs00180489_m1 | myeloproliferativeleukemia virus oncogene |
| BCL2L1-Hs00236329_m1 | BCL2-like 1 |
| GATA1-Hs01085823_m1 | GATA binding protein 1 (globin transcription factor 1) |
| GATA3-Hs00231122_m1 | GATA binding protein 3 |
| PAX5-Hs00172003_m1 | paired box 5 (maturation and commitment of T and B lineages) |
| PRKCZ-Hs00177051_m1 | protein kinase C, zeta |
| IKZF1-Hs00958474_m1 | IKAROS family zinc finger 1 (Ikaros) (maturation and commitment of T and B lineages) |
| SPIB-Hs00162150_m1 | Spi-B transcription factor (Spi-1/PU.1 related) |
| SPIC-Hs00745162_s1 | Spi-C transcription factor (Spi-1/PU.1 related) |
| SPI1-Hs02786711_m1 | spleen focus forming virus (SFFV) proviral integration oncogene spi1 |
| ITGA2B-Hs01116228_m1 | integrin, alpha 2b (platelet glycoprotein IIb of IIb/IIIa complex, antigen CD41) |
| PF4-Hs00427220_g1 | platelet factor 4 |
| ID3-Hs00954037_g1 | inhibitor of DNA binding 3, dominant negative helix-loop-helix protein (neoangiogenesis, endothelial-mesenchymal Transition) |
| ID2-Hs04187239_m1 | inhibitor of DNA binding 2, dominant negative helix-loop-helix protein |
| NFIL3-Hs00705412_s1 | nuclear factor, interleukin 3 regulated (maturation and commitment of T and B lineages) |
| ETS1-Hs00428293_m1 | v-etserythroblastosis virus E26 oncogene homolog 1 (avian) (neoangiogenesis, endothelial-mesenchymal Transition) |
| IL15RA-Hs00542604_m1 | interleukin 15 receptor, alpha |
| TBX21-Hs00203436_m1 | T-box 21 |
| CDKN2A-Hs00923894_m1 | cyclin-dependent kinase inhibitor 2A |
| CDKN2B-Hs00793225_m1 | cyclin-dependent kinase inhibitor 2B (p15, inhibits CDK4) |
| DLL1-Hs00194509_m1 | delta-like 1 (Drosophila) |
| DLL3-Hs01085096_m1 | delta-like 3 (Drosophila) |
| DLL4-Hs00184092_m1 | delta-like 4 (Drosophila) |
| MEIS1-Hs01017441_m1 | Meishomeobox 1 |
| PDK1-Hs01561850_m1 | pyruvate dehydrogenase kinase, isozyme 1 |
| PDK2-Hs00176865_m1 | pyruvate dehydrogenase kinase, isozyme 2 |
| PDK3-Hs00178440_m1 | pyruvate dehydrogenase kinase, isozyme 3 |
| PDK4-Hs01037712_m1 | pyruvate dehydrogenase kinase, isozyme 4 |
| AKT1-Hs00178289_m1 | v-akt murine thymoma viral oncogene homolog 1 |
| PTGER2-Hs04183523_m1 | prostaglandin E receptor 2 (subtype EP2), 53kDa |
| SHB-Hs00182370_m1 | Src homology 2 domain containing adaptor protein B |
| MTOR-Hs00234508_m1 | mechanistic target of rapamycin (serine/threonine kinase) |
| CDX1-Hs00156451_m1 | caudal type homeobox 1 |
| CDX2-Hs01078080_m1 | caudal type homeobox 2 |
| CDX4-Hs00193194_m1 | caudal type homeobox 4 |
| TBPL1-Hs00191595_m1 | TBP-like 1 (ES development, control of transcription) |
| IL6-Hs00985639_m1 | interleukin 6 (interferon, beta 2) |
| PIWIL1-Hs01041737_m1 | piwi-like 1 (Drosophila) |
| SIRT1-Hs01009005_m1 | sirtuin 1 |
| SIRT7-Hs01034735_m1 | sirtuin 7 |
| SOX17-Hs00751752_s1 | SRY (sex determining region Y)-box 17 |
| TAL1-Hs01097987_m1 | T-cell acute lymphocytic leukemia 1 |
| GATA2-Hs00231119_m1 | GATA binding protein 2 |
| PRDM1-Hs00153357_m1 | PR domain containing 1, with ZNF domain (maturation and commitment of T and B lineages) |
| PROM1-Hs01009250_m1 | prominin 1 |
| KIT-Hs00174029_m1 | v-kit Hardy-Zuckerman 4 feline sarcoma viral oncogene homolog |
| CSF2-Hs00929873_m1 | colony stimulating factor 2 (granulocyte-macrophage) |
| FGF4-Hs00999691_m1 | fibroblast growth factor 4 |
| SETD1A-Hs00322315_m1 | SET domain containing 1A |
| SETD2-Hs01014784_m1 | SET domain containing 2 |
| DPPA4-Hs00216968_m1 | developmental pluripotency associated 4 |
| DPPA3-Hs01931905_g1 | developmental pluripotency associated 3 |
| DPPA5-Hs00988349_g1 | developmental pluripotency associated 5 |
| HDAC1-Hs02621185_s1 | histone deacetylase 1 |
| HGF-Hs00300159_m1 | hepatocyte growth factor (hepapoietin A; scatter factor) |
| HDAC4-Hs01041638_m1 | histone deacetylase 4 |
| HDAC7-Hs00248789_m1 | histone deacetylase 7 |
| HDAC9-Hs00206843_m1 | histone deacetylase 9 |
| JUNB-Hs00357891_s1 | jun B proto-oncogene |
| ZFP42-Hs01938187_s1 | zinc finger protein 42 homolog (mouse) |
